# What does scientometry tell us about mercury toxicology and its biological impairments?

**DOI:** 10.1016/j.heliyon.2024.e27526

**Published:** 2024-03-19

**Authors:** Daiane Claydes Baia-da-Silva, Paulo Fernando Santos Mendes, Diane Cleydes Baia da Silva, Victória Santos Chemelo, Leonardo Oliveira Bittencourt, Pedro Magalhães Padilha, Reinaldo Barreto Oriá, Michael Aschner, Rafael Rodrigues Lima

**Affiliations:** a Laboratory of Functional and Structural Biology, Institute of Biological Sciences, Federal University of Pará, Belém, PA, Brazil; b School of Veterinary Medicine and Animal Science, Institute of Biosciences, São Paulo State University, Botucatu, SP, Brazil; c Laboratory of Tissue Healing, Ontogeny and Nutrition, Department of Morphology, School of Medicine, Institute of Biomedicine, Federal University of Ceara, Fortaleza, CE, Brazil; d Department of Molecular Pharmacology, Albert Einstein College of Medicine, Bronx, NY, USA

**Keywords:** Bibliometric analysis, Health, Mercury, Methylmercury, Mercuric dichloride, Neurotoxicology

## Abstract

Mercury is a toxic pollutant that poses risks to both human and environmental health, making it a pressing public health concern. This study aimed to summarize the knowledge on mercury toxicology and the biological impairments caused by exposure to mercury in experimental studies and/or diagnosis in humans. The research was conducted on the main collection of Web of Science, employing as a methodological tool a bibliometric analysis. The selected articles were analyzed, and extracted data such as publication year, journal, author, title, number of citations, corresponding author's country, keywords, and the knowledge mapping was performed about the type of study, chemical form of mercury, exposure period, origin of exposure, tissue/fluid of exposure measurement, mercury concentration, evaluation period (age), mercury effect, model experiments, dose, exposure pathway, and time of exposure. The selected articles were published between 1965 and 2021, with Clarkson TW being the most cited author who has also published the most articles. A total of 38% of the publications were from the USA. These studies assessed the prenatal and postnatal effects of mercury, emphasizing the impact of methylmercury on neurodevelopment, including motor and cognitive evaluations, the association between mercury and autism, and an evaluation of its protective effects against mercury toxicity. In observational studies, the blood, umbilical cord, and hair were the most frequently used for measuring mercury levels. Our data analysis reveals that mercury neurotoxicology has been extensively explored, but the association among the outcomes evaluated in experimental studies has yet to be strengthened. Providing metric evidence on what is unexplored allows for new studies that may help governmental and non-governmental organizations develop guidelines and policies.

## Highlights

• Study maps the toxicological effects of exposure to mercury.
• The effects in the nervous and cardiovascular systems to mercury were mapped.
• The exposure to methylmercury promotes changes in the central nervous system.
• Oxidative stress was the main damage mechanism investigated.
• The main route of exposure/intoxication to mercury is food.

## 1 Introduction

Mercury is a toxic pollutant that poses a risk to both human and environmental health [1,2]. This metal is widely found in nature as a result of natural factors, such as volcanic upwellings, and anthropogenic sources, such as coal-fired power plant activities, artisanal gold mining, ferrous metal manufacturing, cement production, waste incineration, and chlorine and caustic soda production [3]. It occurs in three oxidation states Hg^+^ and Hg^2+^ species also form inorganic compounds such as mercury I chloride (Hg_2_Cl_2_) and mercury II chloride (HgCl_2_), whereas Hg^2+^ ions form organic species such as methylmercury (MeHg^+^) [[4], [5]].

Exposure to mercury poses a significant public health concern, and potential toxic effects have been observed since the late 17th century when Huguenot hat makers used mercury nitrate in the treatment of felt for hat production [6]. Additional concerns emerged regarding the use of mercury fulminate as an explosive detonator, and more recently, dental amalgam and ethylmercury (Ethyl(2-mercaptobenzoato-(2-)-O,S) mercurate(1-) sodium, or thimerosal) have raised worries as vaccine preservatives [6]. Public health risks secondary to mercury contamination were discovered in 1950 when mercury waste was released into the Minamata Basin, Japan, causing a neurological syndrome (called Minamata disease) in the local population that consumed fish contaminated with mercury [[7], [8], [9], [10], [11]]. Today, health concerns are focused in the Amazon, which has high rates of mercury release into the air due to fires and artisanal mining activities, as well as vulnerable riverside populations whose diets are based on the consumption of mercury-contaminated fish [[12], [13], [14], [15]].

Mercury exposure is associated with alterations in the central nervous system (neuropsychological dysfunctions such as memory, cognition, visuospatial, and motor deficits), cardiovascular (arrhythmias and cardiomyopathies), reproductive, renal, and endocrine systems [1,7,[16], [17], [18], [19]]. Recent experimental studies have demonstrated the effects of mercury on organs, tissues, and cells that had not yet been studied as targets, such as the salivary gland [[20], [21], [22], [23]], alveolar bone [[24], [25], [26]], dental pulp [27], and dental enamel [28]. This indicates that mercury affects more body regions than was previously believed.

Considering that mercury undergoes a biomagnification throughout trophic levels and that exposure to mercury occurs through food consumption [15,29,30], understanding the harmful effects of exposure to mercury creates a fertile field for further research to gain insights and provides valuable insights into how mercury damages cells and tissues, improving our understanding of its toxicity and its impact of health.

Therefore, this work aimed to map research related to mercury toxicology and the biological impairments caused by mercury. Additionally, this study aimed to assess existing gaps in knowledge, identify researchers and relevant lines of investigation within the scientific community, and explore potential associations of mercury with environmental disasters.

## 2 Materials and methods

### 2.1 Research strategy and selection of studies

In this investigation, a search was conducted in March 2023 on the main collection of the Web of Science database (WoS-CC) by two independent examiners using a search key (Table 1), with no language or year restrictions. The methodology followed the approach described by de Souza Né et al. (2023) [31], using bibliometric analysis as a methodological tool. Highly cited articles significantly influence the generation and dissemination of scientific knowledge, as they serve as a basis and shape the further research in clinical practice [[32], [33], [34], [35]].

**Table 1:** Research strategy.

Database	Research strategy
Web of Science- Core Collection	TS = (“atmospheric mercury” OR “dietary methylmercury” OR “diethyl Hg” OR “diethyl mercury” OR “diethyl-mercury” OR diethylmercury OR “di-ethylmercury” OR “ethyl Hg” OR “ethyl mercury” OR “ethyl-mercury” OR ethylmercury OR monoethylmercury OR “monomethyl mercury” OR “ethyl mercury-chloride” OR “ethyl-mercury-chloride” OR “methoxy ethyl mercury chloride” OR “methoxy-ethyl-mercury chloride” OR “methoxy-ethyl-mercury-chloride” OR “dimethyl Hg” OR “dimethyl-mercury” OR “dimethyl mercury” OR “mercury dimethyl” OR dimethylmercury OR “methl Hg” OR “methyl mercury” OR “methyl-mercury” OR methylmercury OR monomethylmercury OR “monomethyl mercury” OR “methyl mercuric-chloride” OR “methyl-mercury-chloride” OR “elemental mercury” OR “metallic mercury” OR mercury OR “mercury compound cinnabar” OR “mercuric triflate” OR “organomercurial” OR “organomercury” OR “phenyl mercury” OR phenylmercury OR “phenyl-mercury”) AND TS = (disease OR Health OR Toxicology OR Toxic OR toxicity OR poisoning OR intoxication OR exposure OR exposition OR environmental OR pollutant OR pollution OR emission OR environment).

The search results were organized in descending order of the number of citations, and the 100 most cited articles were selected. Articles that addressed the damage caused by mercury in animal experiments and/or diagnosis in humans included studies on the mechanisms, biomarkers, and biochemical and/or tissue effects were selected. Articles that investigated only the biogeochemical cycle (including oxidation processes in the atmosphere, land-atmosphere and ocean-atmosphere cycling, and methylation processes in the ocean), toxicological effects on plants and/or soil, microorganisms, geology, photoremediation, biomonitoring, water analysis, letters, comments, editorials, and abstracts from conferences or congresses were excluded. Disagreements between evaluators were resolved through discussion and consensus building.

### 2.2 Data extraction of bibliometric parameters

The selected articles were read in full, and the following data were extracted: journal, author, keywords, title, publication year, number of citations in the main Web of Science collection, citation density (mean based on the ratio of the numbers of citations and the period since the year of publication), country and continent of the corresponding author, and DOI/URL. To compare number of citations, the selected articles were searched using Scopus and Google Scholar. The ranking of articles was determined based on the number of citations in the WoS-CC, with citation density used as a tiebreaker when necessary.

### 2.3 Data analysis

Descriptive analysis of the data was performed using Microsoft Excel 2019. Graphical representation of the origin of the articles (country and continent) was performed using MapChart (https://mapchart.net/). Authorship and keyword networks were generated using Visualization of Similarities Viewer (VOSviewer) software. This software organizes the data into clusters, with node size reflecting the number of articles and line thickness denoting the strength of the interaction between terms [36].

### 2.4 Content analysis

The following related data were extracted from the articles: the type of study, chemical form of mercury, frequency of exposure period, source of exposure, tissue/fluid of exposure measures, mercury concentration, frequency of evaluation period, georeferencing of exposed populations, mercury effect, model experiments and animal, dose, exposure pathway, and time of exposure. Studies were classified according to study design into literature reviews, experimental (in vitro, in vivo, in situ, and ex vivo) studies, case reports, case series, cross-sectional studies, case-control studies, and cohort studies.

## 3 Results

### 3.1 Selected studies and bibliometric analysis

**Fig. 1:**
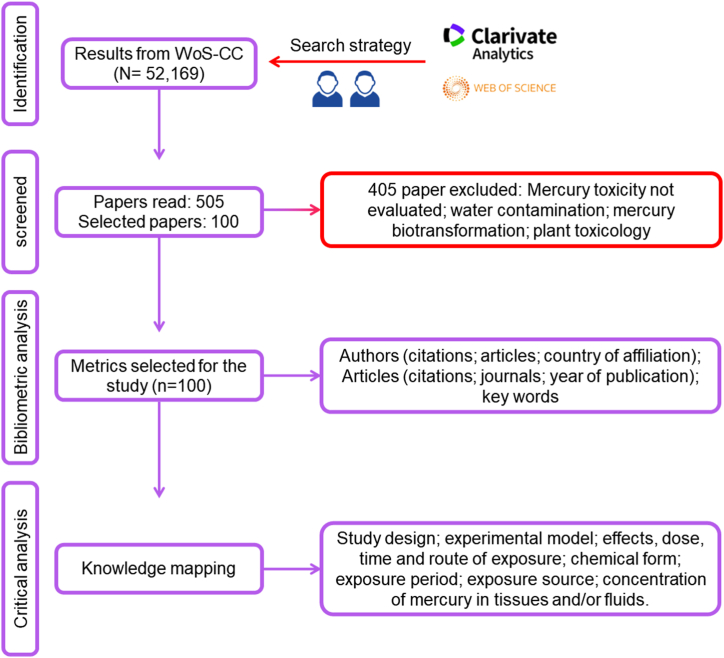
Article search and selection process.

The search strategy retrieved 52,169 results, of which 505 were evaluated. A total of 405 articles were excluded for not evaluating the toxicological effects of mercury and/or for dealing with water contamination and/or biotransformation or for evaluating toxicological effects in plants. Only the 100 most-cited articles that met the selection criteria were included (Fig. 1).

The selected articles cited a total of 61,747 articles in WoS-CC, and citation density ranged from 177.48 to 5.41. The most cited article and with the highest citation density was “Hazards of heavy metal contamination” by Jarup (2003) [37], with 3727 citations and a citation density of 177.48, a review of literature on the toxicity of mercury in its different chemical forms. The number of citations for Google and Scopus was higher than that for WoS-CC (Table 2).

**Table 2:** The 100 most cited articles about the toxic effect of mercury.

R^a^	Author, Years	Description	Number of citations			DOI or URL
			WoS-CC^b^ (Citation density^c^)	Google	Scopus	
1	Jarup, 2003[37]	Reviews the occurrence, exposure, dose, and health effects of mercury	3727 (177.48)	7578	4262	10.1093/bmb/ldg032
2	Valko et al., 2005 [110]	Reviews mercury toxicity and oxidative stress mechanism	3264 (171.79)	5776	3647	10.2174/0929867053764635
3	Stohs and Bagchi, 1995 [111]	Reviews oxidative mechanisms and mercury damage	3207 (110.59)	5361	3555	10.1016/0891-5849(94)00159-H
4	Rice and Barone, 2000 [92]	Reviews the effect of methylmercury during the critical period of neurodevelopment	2022 (84.25)	3265	2209	10.1289/ehp.00108s3511
5	Markesbery, 1997 [112]	Reviews oxidative stress in Alzheimer's disease and the role of mercury in inducing this process	1871 (69.30)	2902	1980	10.1016/S0891-5849(96)00629-6
6	Lushchak, 2011 [113]	Reviews mercury-induced oxidative stress in aquatic animals	1566 (120.46)	2265	1669	10.1016/j.aquatox.2010.10.006
7	Clarkson and Magos, 2006 [6]	Reviews mercury toxicity and protective mechanisms	1513 (84.06)	2358	1639	10.1080/10408440600845619
8	Boening, 2000 [114]	Reviews mercury toxicity in aquatic animals, amphibians, birds, terrestrial invertebrates, and mammals	1466 (61.08)	2249	1556	10.1016/S0045-6535(99)00283-0
9	Mozaffarian and Rimm, 2006 [3]	Reviews the risks and benefits for human health of consuming mercury-contaminated fish and shellfish	1382 (76.78)	2459	1545	10.1001/jama.296.15.1885
10	Harada, 1995 [8]	Reviews methylmercury poisoning in Japan and Minamata disease	1353 (46.66)	2275	1475	10.3109/10408449509089885
11	Grandjean and Landrigan, 2006 [52]	Reviews the neurotoxicity of methylmercury	1334 (74.11)	2172	1470	10.1016/S0140-6736(06)69665-7
12	Driscoll et al., 2013 [115]	Reviews the emission sources and effects of mercury on human and wild animal health, and the impact of policies to control this metal	1319 (119.91)	1806	1392	10.1021/es305071v
13	Clarkson et al., 2003 [44]	Reviews the toxicity of mercury, focusing on the form of exposure and clinical manifestations	1290 (61.43)	1995	1454	10.1056/NEJMra022471
14	Grandjean et al., 1997 [16]	Cohort study evaluating neurobehavioral effects in children exposed to methylmercury during the prenatal period	1216 (45.04)	2128	1356	10.1016/S0892-0362(97)00097-4
15	Bakir et al., 1973 [49]	Reviews the epidemiology of methylmercury intoxication in Iraq and the clinical manifestations in fetuses and infants	1162 (22.78)	2102	1196	10.1126/science.181.4096.230
16	Tchounwou et al., 2003 [1]	Reviews human exposure to mercury and its health effects and dietary influence on toxicity	1021 (48.62)	1377	1087	10.1002/tox.10116
17	Grandjean and Landrigan, 2014 [53]	Reviews methylmercury neurotoxicity and neurobehavioral effects	1012 (101.20)	1634	1107	10.1016/S1474-4422(13)70278-3
18	Mergler et al., 2007 [116]	Review the effects of methylmercury exposure on human health: Neurological, cardiovascular, reproductive, and immunological outcomes, co-contaminants, and risk assessment	874 (51.41)	1337	907	10.1579/0044-7447(2007)36[3:MEAHEI]2.0.CO;2
19	Zahir et al., 2005 [117]	Reviews the exposure to low doses of mercury and risk to human health: neurological, renal, immunological, cardiovascular, and genomic effects	783 (41.21)	1227	877	10.1016/j.etap.2005.03.007
20	Clarkson, 2002 [46]	Reviews the human exposure and toxicology of mercury vapor methylmercury and ethylmercury in the thimerosal form	729 (33.14)	1304	818	10.1289/ehp.02110s111
21	Scheulhammer et al., 2007 [118]	Reviews the effects of methylmercury in wild birds, mammals, and fish	712 (41.88)	1122	750	10.1579/0044-7447(2007)36[12:EOEMOT]2.0.CO;2
22	Renzoni et al., 1998 [119]	Reviews the health risks arising from the consumption of mercury-contaminated fish and seafood	682 (26.23)	861	708	10.1006/enrs.1998.3832
23	Scheuhammer, 1987 [120]	Reviews the chronic toxicity of inorganic mercury and methylmercury in poultry	653 (17.65)	1024	704	10.1016/0269-7491(87)90173-4
24	Wolfe et al., 1998 [121]	Reviews mercury toxicity in mammals and poultry	645 (24.81)	1023	683	10.1002/etc.5620170203
25	Clarkson, 1997 [45]	Reviews the toxicology of mercury, the form of contamination, and effects of exposure.	612 (22.67)	1065	690	10.3109/10408369708998098
26	Baccarelli and Bollati, 2009 [122]	Reviews epigenetic alterations by methylmercury	592 (39.47)	981	676	10.1097/MOP.0b013e32832925cc
27	Flora et al., 2008 [123]	Reviews the oxidative stress generated by mercury and the reversal with chelation therapy	590 (36.88)	1317	719	https://journals.lww.com/ijmr/Abstract/2008/28040/Heavy_metal_induced_oxidative_stress___its.12.aspx
28	Grandjean et al., 1998 [54]	Cohort study evaluating the neurobehavioral effects in children exposed to methylmercury during the prenatal period	568 (21.85)	817	604	10.1006/enrs.1997.3804
29	Bridges and Zalups, 2005 [66]	Reviews mercury transport in target organs, and ionic and molecular mimicry	536 (28.21)	793	581	10.1016/j.taap.2004.09.007
30	Goyer, 1997 [124]	Reviews the protective effect of selenium against the toxic effects of mercury	533 (19.74)	1048	616	10.1146/annurev.nutr.17.1.37
31	Jan et al., 2015 [125]	Reviews the effect of mercury on human health, mechanistic insights of toxicity, and the antioxidant defense system	532 (59.11)	985	625	10.3390/ijms161226183
32	Ganther et al., 1972 [126]	*In vivo* experimental study evaluating the protective effect of selenium against mercury toxicity	531 (10.21)	778	505	10.1126/science.175.4026.1122
33	Guallar et al., 2002 [38]	Case-control evaluating the association between mercury and docosahexaenoic acid levels with the risk of myocardial infarction	517 (23.50)	913	595	10.1056/NEJMoa020157
34	Davidson et al., 1998 [50]	Cohort study evaluating the effect of prenatal and postnatal dietary exposure to methylmercury on neurodevelopment	496 (19.08)	841	571	10.1001/jama.280.8.701
35	Zalups, 2000 [127]	Reviews molecular interactions of mercury in the kidney and mechanisms of absorption, accumulation, elimination, and toxicity	483 (20.13)	789	544	https://pharmrev.aspetjournals.org/content/52/1/113.short
36	Vahter et al., 2007 [128]	Reviews the health effects of mercury across genders	470 (27.65)	708	499	10.1016/j.envres.2006.08.003
37	Rossignol and Frye, 2012b [129]	Systematic review of the ethylmercury-induced mitochondrial dysfunction and its association with autism spectrum disorder	468 (39.00)	779	507	10.1038/mp.2010.136
38	Karagas et al., 2012 [55]	Reviews the exposure to low doses of methylmercury and risk to human health: Neurocognitive, behavioral, cardiovascular, and immune effects	448 (37.33)	655	474	10.1289/ehp.1104494
39	Wu et al., 2016 [130]	Reviews the toxicity and oxidative mechanisms of mercury	434 (54.25)	673	477	10.1007/s11356-016-6333-x
40	Preston et al., 1993 [131]	*In vitro* experimental study that evaluating the replacement of cysteine by serine in the CHIP28 protein, to identify which of the 4 cysteines (87, 102, 152 or 189) is sensitive to mercury	433 (13.97)	703	497	10.1016/S0021-9258(18)54108-9
41	Myers et al., 2003 [51]	Cohort study evaluating the prenatal methylmercury exposure on childhood neurodevelopment of Seychellois children	416 (19.81)	729	487	10.1016/S0140-6736(03)13371-5
42	Castro-Gonzalez and Mendez-Armenta, 2008 [132]	Reviews the effects of mercury on human health and the possible association with fish consumption	411 (25.69)	881	462	10.1016/j.etap.2008.06.001
43	Holmgren and Lu, 2010 [133]	Reviews the role of thioredoxin and thioredoxin reductase in the defense mechanism to oxidative damage in mercury intoxication	407 (29.07)	591	425	10.1016/j.bbrc.2010.03.083
44	Ali et al., 1992 [134]	*In vivo* and in vitro study evaluating the oxidative damage of the organometallic compounds methylmercury and trimethyltin	402 (12.56)	582	441	https://europepmc-org.ez3.periodicos.capes.gov.br/article/med/1475065
45	Salonen et al., 1995 [39]	Cohort study evaluating the relationship of dietary intake of fish and mercury with the lipid peroxidation, risk of acute myocardial infarction, and death from coronary and cardiovascular disease	391 (13.48)	751	446	10.1161/01.CIR.91.3.645
46	Oken et al., 2005 [89]	Cohort study evaluating the impact of fish consumption (with possible mercury contamination) during pregnancy on neurodevelopment	387 (20.37)	636	431	10.1289/ehp.8041
47	Spry and Wiener, 1991 [135]	Review the mercury bioavailability and toxicity to fish in lakes with low alkalinity	371 (11.24)	675	397	10.1016/0269-7491(91)90034-T
48	Carvalho et al., 2008 [4]	*In vitro* experimental study evaluating the effects of mercury chloride and monomethylmercury on thioredoxin system proteins in mammals	363 (22.69)	521	384	10.1074/jbc.M710133200
49	Zheng et al., 2003 [136]	Reviews the transport of mercury in the blood-brain barrier and its neurotoxicity	360 (17.14)	542	404	10.1016/S0041-008X(03)00251-5
50	Rahman and Singh, 2019 [137]	Reviews the general mechanisms of mercury toxicity in microorganisms, marine plants, animals, and humans	355 (71.00)	524	384	10.1007/s10661-019-7528-7
51	Chin-Chan et al., 2015 [138]	Reviews the association between mercury and neurodegenerative diseases (Parkinson and Alzheimer)	354 (39.33)	602	384	10.3389/fncel.2015.00124
52	Modabbernia et al., 2017 [139]	Review on the environmental exposure to mercury and the risk for autism spectrum disorder	349 (49.86)	624	372	10.1186/s13229-017-0121-4
53	Debes et al., 2006a [56]	Cohort study evaluating the impact of prenatal methylmercury exposure on neurobehavior	347 (19.28)	570	237	10.1016/j.ntt.2006.02.005
54	Debes et al., 2006b [57]	Cohort study evaluating the impact of prenatal methylmercury exposure on neurobehavior	347 (19.28)	570	159	10.1016/j.ntt.2006.02.004
55	Counter and Buchanan, 2004 [140]	Reviews epidemiologic studies of methylmercury exposure in children	346 (17.30)	633	402	10.1016/j.taap.2003.11.032
56	Gorell et al., 1999 [141]	Case-control evaluating the occupational exposure to mercury as a risk factor for Parkinson's disease	344 (13.76)	553	375	https://europepmc.org/article/med/10385887
57	Farina et al., 2013 [142]	Reviews the effects of mercury and neurodegenerative mechanisms.	340 (30.91)	516	378	10.1016/j.neuint.2012.12.006
58	Zhang and Wong, 2007 [143]	Reviews the environmental mercury in China: Sources of contamination, ecological contaminants, routes of exposure, and risks to human health	338 (19.88)	549	365	10.1016/j.envint.2006.06.022
59	Matsumoto et al., 1965 [87]	Case report of the neurological impacts of prenatal mercury poisoning, fetal Minamata disease	336 (5.69)	436	306	10.1097/00005072-196510000-00002
60	Gorell et al., 1997 [144]	Case-control evaluating the association between Parkinson's disease and occupational exposure to mercury	333 (12.33)	562	383	10.1212/WNL.48.3.650
61	Rossignol and Frye, 2012a [145]	Reviews the mechanisms associated with the development of autism spectrum disorder and exposure to mercury	332 (27.67)	575	354	10.1038/mp.2011.165
62	Bose-O’Reilly et al., 2010 [146]	Reviews exposure to mercury and health risk in children	331 (23.64)	646	370	10.1016/j.cppeds.2010.07.002
63	Clarkson, 1993 [48]	Reviews pathways of human exposure, methylmercury deposition, toxicity, and dose-response exposure	329 (10.61)	672	442	10.2307/3431518
64	Ralston and Raymond, 2010 [147]	Reviews the protective effect of selenium against damage induced by methylmercury	328 (23.43)	491	354	10.1016/j.tox.2010.06.004
65	Gochfeld, 2003 [148]	Review of six cases of exposure to different species of mercury, bioavailability, and absorption of mercury	326 (15.52)	646	381	10.1016/S0147-6513(03)00060-5
66	Bosch et al., 2016 [149]	Reviews the human health risk of dietary exposure to mercury-contaminated fish	319 (39.88)	518	349	10.1002/jsfa.7360
67	Barboza et al., 2018 [150]	*In vivo* experimental study evaluating the toxic effects of microplastics and mercury in fish: Neurotoxicity and oxidative damage	317 (52.83)	457	340	10.1016/j.aquatox.2017.12.008
68	Finkelman et al., 2002 [151]	Reviews health problems caused and/or aggravated by environmental mercury contamination, and by inhaling coal Dust	317 (14.41)	530	370	10.1016/S0166-5162(02)00125-8
69	Oken et al., 2008 [152]	Cohort study evaluating the effect of prenatal exposure to mercury and the association with neurodevelopmental changes	313 (19.56)	524	345	10.1093/aje/kwn034
70	Choi et al., 1978 [153]	Case reports of prenatal methylmercury intoxication and cerebral alterations in newborns	311 (6.76)	446	322	10.1097/00005072-197811000-00001
71	Iavicoli et al., 2009 [154]	Reviews the effects of mercury on endocrine system modulation and its effects on health	306 (20.40)	507	336	10.1080/10937400902902062
72	Amin-Zaki et al., 1974 [155]	Cross-sectional study with clinical and laboratory evaluation of the effects of exposure to methylmercury resulting from a disaster in rural Iraq	305 (6.10)	543	293	https://doi-org.ez3.periodicos.capes.gov.br/10.1542/peds.54.5.587
73	Farina et al., 2011[93]	Reviews the molecular mechanisms of methylmercury-induced neurotoxicity	298 (22.92)	450	314	10.1016/j.lfs.2011.05.019
74	Wigle et al., 2008 [11]	Reviews the association between prenatal and/or early life exposure to mercury and fetal, infant, and adult health	296 (18.50)	541	337	10.1080/10937400801921320
75	Evers et al., 2008 [156]	*In vivo* experimental study evaluating the methylmercury toxicity in *Gavia immer* and the potential impacts on behavior, physiology, survival, and reproductive success	293 (18.31)	442	306	10.1007/s10646-007-0168-7
76	Robertson and Orrenius, 2000 [157]	Reviews mechanisms involved in activating mercury-induced apoptosis	292 (12.17)	455	314	10.1080/10408440008951122
77	Holmes et al., 2009 [158]	Reviews epidemiological evidence on the effects of prolonged low-level exposure to various forms of mercury on the renal, cardiac, immune, and nervous systems	289 (19.27)	514	324	10.1016/j.scitotenv.2009.09.043
78	Peraza et al., 1998 [159]	Reviews the effects of micronutrients against mercury toxicity	285 (10.96)	533	326	10.2307/3433921
79	Trasande et al., 2005 [160]	Cohort study evaluating the association between exposure to methylmercury and loss of intelligence quotient	284 (14.95)	544	333	10.1289/ehp.7743
80	Rodier, 1995 [161]	Reviews the risk of mercury toxicity during neurodevelopment	283 (9.76)	496	332	10.2307/3432351
81	Windham et al., 2006 [162]	Case-control evaluating the association between autism spectrum disorders and exposure to mercury	280 (15.56)	515	320	10.1289/ehp.9120
82	Beckers and Rinklebe, 2017 [163]	Reviews the behavior of mercury in soils and sediments, as well as implications for human health	275 (39.29)	371	312	10.1080/10643389.2017.1326277
83	Elia et al., 2003 [86]	*In vivo* experimental study evaluating the antioxidant response to exposure to mercury	275 (13.10)	419	295	10.1016/S0147-6513(02)00123-9
84	Balali-Mood et al., 2021 [34]	Reviews mechanisms of mercury toxicity in animals and humans	274 (91.33)	486	309	10.3389/fphar.2021.643972
85	Harada, 1978 [9]	Reviews congenital Minamata disease triggered by intrauterine exposure to methylmercury	272 (5.91)	471	291	10.1002/tera.1420180216
86	Steuerwald et al., 2000 [58]	Cohort study evaluating the prenatal exposure to methylmercury and changes in neurodevelopment	271 (11.29)	485	312	10.1067/mpd.2000.102774
87	Martin and Fry, 2018 [164]	Reviews the relationship between DNA methylation and mercury exposure	269 (44.83)	390	292	10.1146/annurev-publhealth-040617-014629
88	Rossignol et al., 2014 [165]	Systematic review on the associations between prenatal and childhood exposure to methylmercury and autism spectrum disorder	267 (26.70)	460	302	10.1038/tp.2014.4
89	Kosta et al., 1975 [166]	Reviews of the protective capacity of selenium against inorganic mercury toxicity	265 (5.41)	358	266	10.1038/254238a0
90	Fitzgerald and Clarkson, 1991 [47]	Reviews methylmercury exposure with a focusing on atmospheric cycle deposition, marine biogeochemical, human exposure, and health risks	264 (8.00)	479	288	10.2307/3431225
91	Taylor et al., 2014 [167]	Reviews the association between mercury used in vaccines and autism spectrum disorder	263 (26.30)	771	311	10.1016/j.vaccine.2014.04.085
92	Syversen and Kaur, 2012 [168]	Reviews the toxicology of inorganic mercury and the neurotoxicology of methylmercury	262 (21.83)	404	280	10.1016/j.jtemb.2012.02.004
93	Rooney, 2007 [169]	Reviews the role of thiols, dithiols, and nutritional factors resulting from mercury poisoning	262 (15.41)	397	285	10.1016/j.tox.2007.02.016
94	Rana, 2008 [170]	Reviews mercury-induced apoptotic mechanisms	260 (16.25)	406	289	10.1016/j.jtemb.2008.08.002
95	Daniels et al., 2004 [171]	Cohort study evaluating the mercury-containing fish intake during the prenatal period and effects on neurodevelopment	255 (12.75)	437	284	10.1097/01.ede.0000129514.46451.ce
96	Grandjean et al., 2001 [59]	Cohort study evaluating the neurobehavioral deficit associated with prenatal exposure to polychlorinated biphenyls to seafood neurotoxicants	254 (11.04)	418	306	10.1016/S0892-0362(01)00155-6
97	Kim et al., 2016 [172]	Reviews impacts of mercury on human health: neurological, renal, cardiovascular, reproductive, and genetic-epigenetic effects	248 (31.00)	361	270	10.1016/j.jhazmat.2015.11.031
98	Castoldi et al., 2001 [173]	Reviews the neurotoxicity and molecular effects of exposure to low doses of methylmercury	247 (10.74)	432	290	10.1016/S0361-9230(01)00458-0
99	Houston, 2011 [174]	Reviews mercury toxicity associated with cardiovascular changes and their clinical consequences	244 (18.77)	428	268	10.1111/j.1751-7176.2011.00489.x
100	Landrigan, 2010 [175]	Reviews the association of autism spectrum disorder with environmental exposures to methylmercury	243 (17.36)	627	285	10.1097/MOP.0b013e328336eb9a

a R: rank.

b WoS-CC: Web of Science Core Collection.

c Citation density: Mean based on the ratio of the numbers of citations and the period since the year of publication up to March 2023.

#### 3.1.1 Period of publication

**Fig. 2:**
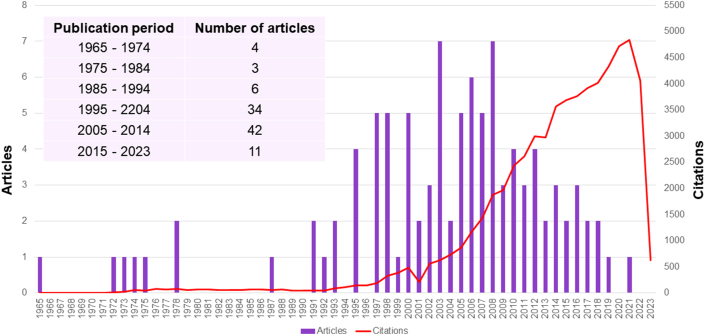
Publication and citation trends per year of selected articles.

The 100 most cited articles were published between 1965 and 2021. In the first 30 years (1965–1994), only 13 of the articles were published, with the number of publications increasing over time (e.g., 2005–2014; n = 42 articles) (Fig. 2).

#### 3.1.2 Journal of publication

**Fig. 3:**
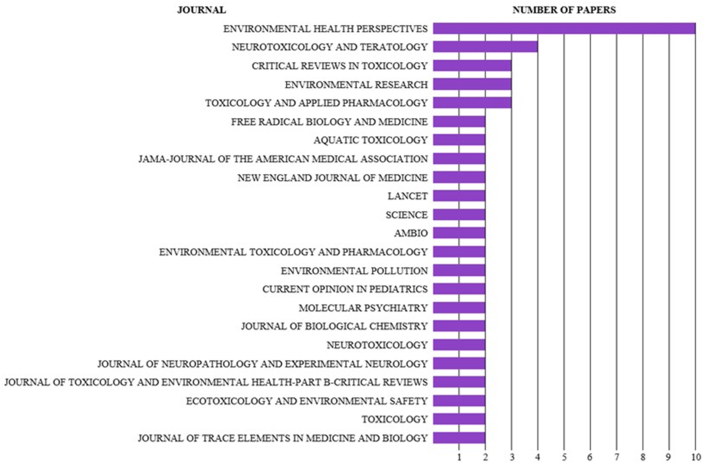
Journal featuring a minimum of two published articles.

The selected articles were published in 64 journals. The journals with the highest number of publications were Environmental Health Perspectives (15.38%; 5311 citations), Neurotoxicology and Teratology (6.15%; 2164 citations), Critical Reviews in Toxicology (4.61%; 3158 citations), Environmental Research (4.61%; 1720 citations), and Toxicology and Applied Pharmacology (4.61%; 1242 citations) (Fig. 3). Among the journals, 27.70% published two articles and 63.07% published only one article.

#### 3.1.3 Author contributions

**Fig. 4:**
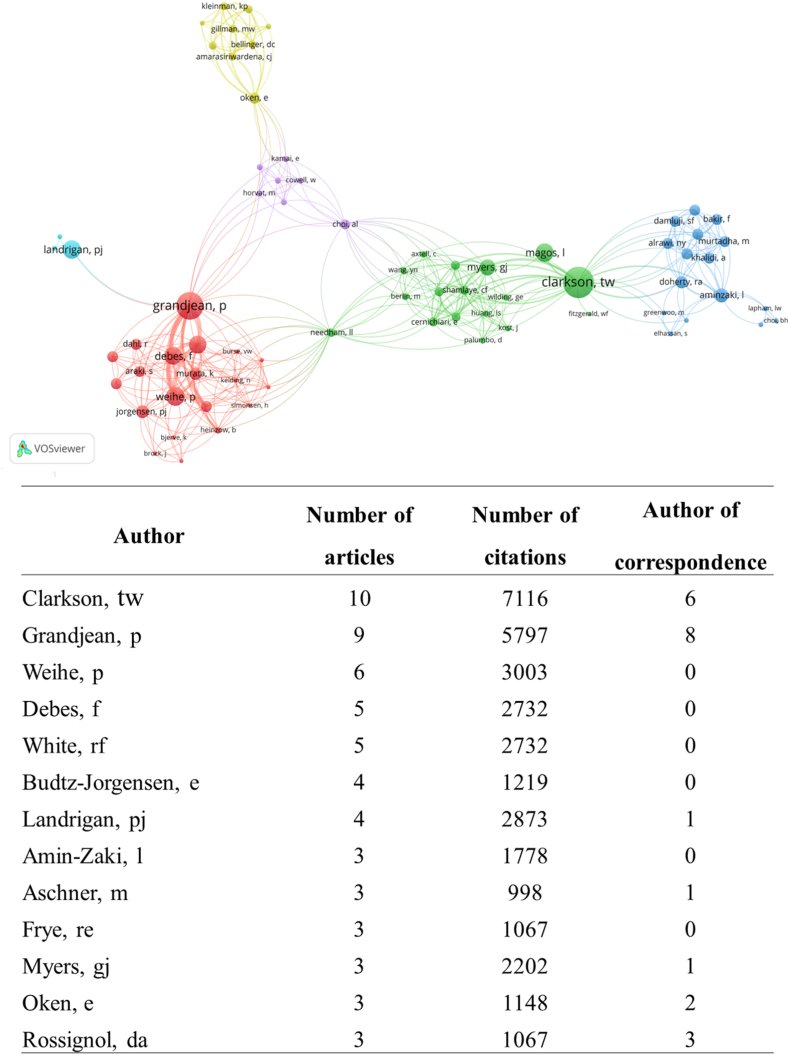
Network showing the largest set of connected items of coauthor contribution and list of authors with at least three articles. The node size represents the number of articles from each author.

A total of 318 authors contributed to selected articles. One author published ten articles (0.31%), one published nine articles (0.31%), one published six articles (0.31%), two authors published five articles (0.62%), two published four articles (0.62%), six published three articles (1.88%), six published three articles (1.88), 36 published two articles (11.32%), and 269 authors published only one article (84.59%) (Fig. 4). The authors with the highest number of publications and citations in the WoC-CC were Clarkson TW (n = 10 articles; 7116 citations) and Grandjean P (n = 9 articles; 5797 citations) (Fig. 4).

Other authors with a high number of citations were Jarup L (3727 citations), Cronin MTD (3264 citations), Morris H (3264 citations), Volko M (3264 citations), Bagchi D (3207 citations), Stohs SJ (3207 citations), all with just one article.

#### 3.1.4 Keywords

**Fig. 5:**
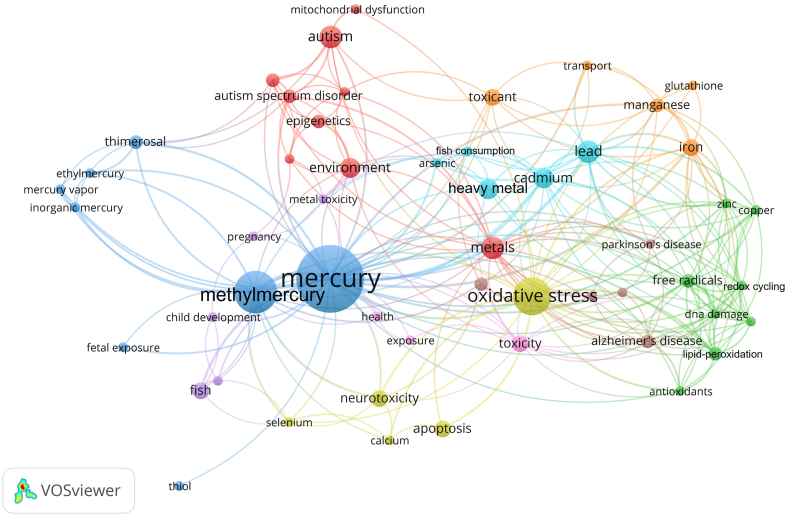
Network of author keywords with a minimum of two occurrences. The size of each node corresponds to the keyword's frequency. Larger nodes indicate greater frequency, and colors differentiate clusters. (For interpretation of the references to color in this figure legend, the reader is referred to the Web version of this article.)

A total of 276 author keywords were identified in the selected articles. The most frequently used words were mercury (26 articles), methylmercury (15 articles), and oxidative stress (12 articles) (Fig. 5).

#### 3.1.5 Geographic distribution of scientific knowledge

The corresponding authors of the selected articles resided in 25 countries, comprising 13 European, six Asian, three North American, one South American, one African, and one Oceanian countries. The United States had the highest number of publications (51 %) and citations (32, 673).

**Fig. 6:**
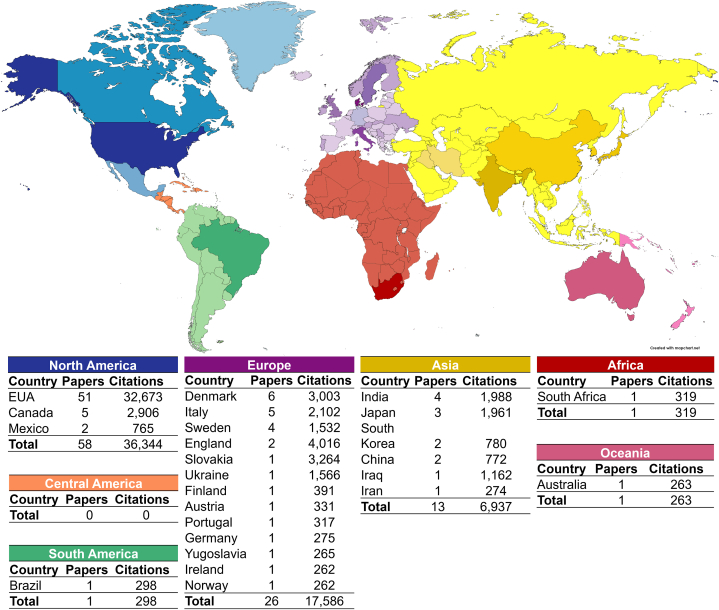
Geographical distribution of the articles on the toxicological effects of mercury selected from the Web of Science Core Collection.

Although most countries were in Europe and Asia, North America had the highest number of publications (58%) and citations (36,344), followed by Europe (26%; 17,586 citations) and Asia (13%; 6937 citations). Some articles were published in South America, Africa, and Oceania, at 1% (298 citations), 1% (319 citations), and 1% (263 citations), respectively. No corresponding authors were from Central America (Fig. 6).

### 3.2 Content analysis

#### 3.2.1 Study design

Most of the selected articles were reviews (73%, in which only 2% were systematic reviews), followed by observational studies (20%) and experimental studies (7%). Among the observational studies, thirteen were cohort studies (5545 citations), four were case controls (1474 citations), two were case reports (647 citations), and one was a cross-sectional study (305 citations). Regarding laboratory studies, four were in vivo (1416 citations), two were in vitro (796 citations), and one was a combined in vivo and in vitro study (402 citations) (Table 3).

**Table 3:** Study design of the top 100 articles cited in WoS-CC regarding the toxic effects of mercury.

Study design	Number of articles	Number of citations
Literature review	73	51,162
Cohort study	13	5545
Case-control	4	1474
*In vivo* experimental study	4	1416
*In vitro* experimental study	2	796
Case report	2	647
*In vitro* and in vivo experimental study	1	402
Cross-sectional study	1	305

#### 3.2.2 Knowledge maps

The selected studies addressed aspects related to the different forms of mercury, route of exposure, dosage, period of exposure, tissue/fluid exposure measures of mercury, period of evaluation, and information regarding damage caused by mercury.

The observational articles evaluated the different chemical forms of mercury; the most studied form was methylmercury (12 articles), and eight articles did not specify the chemical form (Table 4). Seventy-five percent of the studies (15 articles) evaluate the dietary route as a source of human mercury exposure, primarily through the ingestion of contaminated fish (7 articles), followed by seafood (4 articles), bread with wheat contaminated by mercury (3 articles) and whale meat (2 articles). Exposure routes such as inhalation of mercury vapors through occupational exposure and/or polluted air resulting from coal production were investigated in 15.72% (3 articles) of the studies (Table 4).

**Table 4:** Knowledge maps of the observational study.

Author Year	Chemical form of mercury	Exposure period	Source of exposure	Tissue/Fluid of exposure measures	Mercury concentration	Evaluation period (age)/Georeferencing	Mercury effect
Grandjean et al., 1997 [16]	Methylmercury	Prenatal	Feed (whale meat)	Cord blood, children hair, and maternal hair	Total mercury (mean) Maternal hair: 4.27 μg/g; Children hair: 2.99 μg/g; Cord blood: 22.90 μg/L	Children (≅ 7 years old)/Denmark (Faroe Islands)	Neuropsychological dysfunctions primarily involve alterations in the domains of language, attention, and memory, and to a lesser extent, visuospatial and motor functions
Grandjean et al., 1998 [54]	Methylmercury	Prenatal	Feed (whale meat)	Cord blood and maternal hair	Total mercury (mean) Cord blood: 59.00 μg/L; Maternal hair: 12.50 μg/g;	Children (≅ 7 years old)/Denmark (Faroe Islands)	Decreases in brain function, especially in the domains of motor function, language, and memory
Guallar et al., 2002 [38]	Mercury (not specified)	Postnatal	Feed (fish)	Toenail	Total mercury (mean) Toenail: 0.25 μg/g	Adults (≥70 years old)/Finland, Israel, Germany, Scotland, Switzerland, Russia and Spain	Increased risk of myocardial infarction and reduced benefits of fish oil
Davidson et al., 1998 [50]	Methylmercury	Prenatal and postnatal	Feed (fish)	Maternal hair and children hair	Total mercury (mean) Maternal hair: 6.80 μg/g; children hair: 6.50 μg/g	Children (5–7 years old)/Republic of Seychelles	This study does not show changes in language and behavior through prenatal and postnatal exposure to methylmercury from fish consumption
Myers et al., 2003 [51]	Methylmercury	Prenatal	Feed (fish)	Maternal hair	Total mercury (mean) Maternal hair: 6.90 μg/g	Children (9 years old)/Republic of Seychelles	This study did not show changes in neurocognitive, language, memory, motor, perceptual-motor, and behavioral functions due to prenatal exposure to methylmercury from the consumption of oceanic fish
Salonen et al., 1995 [39]	Mercury (not specified)	Postnatal	Feed (fish)	Hair and urine	Total mercury (mean) Hair: 1.92 μg/g; urine: 1.18 μg	Adults (42–60 years old)/Eastern Finland	Associated with risk of acute myocardial infarction, as well as death from coronary heart disease, cardiovascular disease
Oken et al., 2005 [89]	Mercury (not specified)	Prenatal	Feed (fish)	Maternal hair	Total mercury (mean) Maternal hair: 0.55 μg/g	Children (6 months old)/USA	Consumption of fish with higher levels of mercury was associated with lower cognition (assessed by the percent novelty preference on visual recognition memory)
Debes et al., 2006a [56]	Methylmercury	Prenatal	Feed (Seafood)	Cord blood, maternal hair; teenagers' blood and hair	Total mercury (mean) Cord blood: 22.50 μg/L; maternal hair: 4.21 μg/g; teenagers' blood: 4.0 8 μg/L; teenager's hair: 0.96 μg/g	Adolescent (14 years old)/Denmark (Faroe Islands)	Permanent and multifocal effects on brain function, motor, attention and language deficits, and limitation in lexical development
Debes et al., 2006b [57]	Methylmercury	Prenatal	Feed (Seafood)	Cord blood, maternal hair; teenagers' blood and hair	Total mercury (mean) Cord blood: 22.50 μg/L; maternal hair: 4.21 μg/g; teenagers' blood: 4.08 μg/L; teenager's hair: 0.96 μg/g	Adolescent (14 years old)/Denmark (Faroe Islands)	Permanent and multifocal effects on brain function, motor, attention and language deficits, and limitation in lexical development
Gorell et al., 1999 [141]	Mercury (not specified)	Postnatal	Occupational exposures	Not informed	Not informed	Adults (≥50 Years old)/USA (Detroit)	Occupational exposure was not associated with Parkinson's disease
Matsumoto et al., 1965 [87]	Methylmercury	Prenatal	Environmental disaster (Minamata)	Maternal hair; children hair, brain, liver, and kidney	Total mercury Maternal hair: 4.50 μg/g; Children hair (1.4 years old): 35.00 μg/g, children hair (after death): 7.20 μg/g; children brain: 0.03 μg/g, liver: 2.20 μg/g and kidney: 1.90 μg/g	Children (1 years and 4 months old)/Japan (Minamata)	Malnutrition, spastic paralysis of all extremities, pupils unresponsive to light, delay in mental development; Postmortem: asymmetry of the head, diffuse atrophy of the muscles, diminished cerebellum, cellular disorganization of the cortex, hypoplastic brainstem and spinal cord, congestion of the cerebral leptomeninges, reduced corpus callosum, proliferation of microglia.
	Methylmercury	Prenatal	Environmental disaster (Minamata)	Maternal hair; children hair, brain, liver and kidney	Total mercury Maternal hair: 191.00 μg/g; Children hair (3.6 years old): 57.80 μg/g, children hair (after death): 10.01 μg/g; children brain: 0.70 μg/g, liver: 5.70 μg/g and kidney: 11.30 μg/g	Children (3 years and 6 months old)/Japan (Minamata)	Slow growth, mental retardation, malnutrition, severe muscle stiffness, slow nystagmoid; Postmortem: Generalized muscle wasting, edematous and thickened leptomeninges, reduced cerebellum, degeneration, and atrophy of nerve cells in the cortical architecture, reactive proliferation of microglia and oligodendroglia
Gorell et al., 1997 [144]	Mercury (not specified)	Postnatal	Occupational exposures	Not informed	Not informed	Adults (≥50 years old)/USA (Detroit)	Occupational exposure was not associated with Parkinson's disease
Oken et al., 2008 [152]	Mercury (not specified)	Prenatal	Feed (fish)	Maternal blood	Total mercury (mean) Maternal blood: 3.80 ng/g	Children (3 years old)/USA	Cognitive deficit with decreases language functions and in visual motor development (in the domains: visual-spatial, visual-motor, and fine motor skills)
Choi et al., 1978 [153]	Methylmercury	Prenatal	Feed (bread)	Maternal blood; child blood and brain	Total mercury Maternal blood: 726.00 μg/L (on admission)/193.00 μg/L (at delivery); children blood: 516.00 μg/L (at delivery)/575.00 μg/L (at autopsy); Frontal lobe: 1.39 μg/g; parietal lobe: 1.35 μg/g; temporal lobe: 1.02 μg/g; occipital lobe: 1.57 μg/g; calcarine fissure: 1.60 μg/g; basal ganglia: 1.38 μg/g; thalamus: 1.37 μg/g; pons: 1.38 μg/g; medulla: 2.13 μg/g; spinal cord: 1.27 μg/g; optic chiasm: 1.09 μg/g Inorganic mercury Maternal blood: 35.00 μg/L (on admission)/14.00 μg/L (at delivery); children blood: 28.00 μg/L (at delivery)/175.00 μg/L (at autopsy); Frontal lobe: 0.11 μg/g; parietal lobe: 0.26 μg/g; temporal lobe: 0.20 μg/g; occipital lobe: 0.22 μg/g; calcarine fissure: 0.49 μg/g; basal ganglia: 0.27 μg/g; thalamus: 0.18 μg/g; pons: 0.50 μg/g; medulla: 0.80 μg/g; spinal cord: 0.28 μg/g; optic chiasm: 0.52 μg/g	Children (33 days old)/Iraq	Mother: tongue, hands, feet and perioral region numbness, abdominal pain, visual alteration, joint pains, slurred speech, exaggerated deep tendon reflexes in all four extremities. Children: Dead, bilateral pneumonia, disturbance in brain development (incomplete or abnormal migration of neurons to the cerebellar and cerebral cortices, and disturbed cortical organization)
	Methylmercury	Prenatal	Feed (bread)	Maternal blood; Children blood and brain	Total mercury Maternal blood: 1188.00 μg/g (on admission)/748.00 μg/g (at delivery); blood infant: 1.568 μg/L (at delivery)/1726 μg/L (at autopsy); Frontal lobe: 13.70 μg/g, parietal lobe: 7.70 μg/g; temporal lobe: 3.70 μg/g; occipital lobe: 6.20 μg/g; thalamus: 7.40 μg/g; pons: 3.10 μg/g; medulla: 0.40 μg/g; spinal cord: 3.50 μg/g; optic chiasm: 4.70 μg/g; cerebellum: 8.10 μg/g Inorganic mercury Maternal blood: 74 μg/g (on admission)/65 μg/g (at delivery); blood infant: 86 μg/L (at delivery)/98 μg/L (at autopsy); Frontal lobe: 1.10 μg/g, parietal lobe: 0.40 μg/g; temporal lobe: 1.30 μg/g; occipital lobe: 0.80 μg/g; thalamus: 4.30 μg/g; pons: 3.10 μg/g; medulla: 0.20 μg/g; spinal cord: 1.70 μg/g; optic chiasm: 4.10 μg/g; cerebellum: 0.96 μg/g	Children (7 h old)/Iraq	Mother: malaise, dizziness, visual disturbances, numbness and weakness of the limbs and tongue, Unable to see or walk, alteration visual, deep tendon reflexes exaggerated in all four extremities and Babinski responses were present bilaterally Children: Dead, bilateral, disturbance in brain development (incomplete or abnormal migration of neurons to the cerebellar and cerebral cortices, and disturbed cortical organization)
Amin-Zaki et al., 1974 [155]	Methylmercury	Prenatal	Feed (bread)	Maternal blood, milk, and children blood	Total mercury Maternal blood: ≥0.40 μg/g; Children blood: 0.564–4.220 μg/g	Children (6 days–6.5 months old)/Iraq	Mother - malaise, muscle and joint pain, loss of sensitivity in the perioral region and extremities, motor weakness and exaggerated reflexes, visual changes, impaired neurodevelopment. Children- Exaggerated reflexes in the lower extremities, reduced head circumference, changes in muscle tone, impaired motor and mental development, visual changes (blinding, Nystagmus, and strabismus), hearing impairment and cerebral palsy.
Trasande et al., 2005 [160]	Methylmercury	Prenatal	Not informed	Cord blood	Total mercury Cord blood ≥3.50; 4.84; 5.80; 7.13 and 15.00 μg/L	Children (not identified)/USA	Methylmercury concentrations >5.8 μg/L were associated with intelligence coefficient loss. The damage is proportional to mercury levels
Windham et al., 2006 [162]	Mercury (not specified)	Prenatal	Polluted air	Not informed	Not informed	Children (first infancy)/USA (California - San Francisco Bay)	Mercury is associated with autism spectrum disorder
Steuerwald et al., 2000 [58]	Methylmercury	Prenatal	Feed (Seafood)	Cord blood, cord serum and maternal hair	Total mercury (mean) Cord blood: 20.40 μg/g; Cord serum: 2.54 μg/g, Maternal hair: 4.0 8 μg/g	Children (16.8 ± 7.4 days old)/Denmark (Faroe Islands)	Increased risk of neurodevelopmental deficit. Mercury was associated with a decrease in neurological optimality after assessing behavioral skills, most reflexes and responses, and stability of behavioral state during the examination
Daniels et al., 2004 [171]	Mercury (not specified)	Prenatal	Feed (fish)	Umbilical cord tissue	Total mercury (median) Umbilical cord tissue: 0.01 μg/g	Children (15 and 18 months old)/England (Bristol and surrounding areas)	Low mercury concentrations were not related to changes in neurodevelopment (language and communication skills)
Grandjean et al., 2001 [59]	Methylmercury	Prenatal	Feed (Seafood)	Cord blood and umbilical cord tissue	Total mercury (median) Cord blood: 25.70 μg/L	Children (7 years old)/Denmark (Faroe Islands)	Mercury neurotoxicity is increased when co-exposed to polychlorinated biphenyls

These studies evaluated the effects of exposure to mercury during the prenatal (75%; 15 articles), postnatal (20%; 4 articles), and pre and postnatal (5%; 1 article) periods. All studies on postnatal exposure to mercury evaluated damage in adults, whereas the effect of prenatal exposure was evaluated in children (13 articles) and adolescents (2 articles). Populations of the Faroe Islands were evaluated in Denmark (6 articles), the United States (6 articles), the Republic of Seychelles (2 articles), Iraq (2 articles), Japan (1 article), Finland (1 article), England (1 article), and one study evaluated populations in Europe and Asia (Finland, Israel, Germany, Scotland, Switzerland, Russia, and Spain) (Table 4).

Mercury levels were quantified as total mercury and/or inorganic mercury in tissues such as the cord blood, hair, blood, plasma, milk, urine, toenail, brain, liver, and kidney; the concentration of mercury varied according to the tissue or fluid evaluated. Mercury levels in adults were determined in toenail (1 article), hair, and urine (1 article), and two articles did not report the measurement sample/location. The maternal tissues evaluated were hair (9 articles), blood (3 articles), and milk (1 article). Cord blood was evaluated in seven articles, one evaluated cord serum and two evaluated umbilical cord tissue. In children, mercury levels were determined in hair (3 articles), blood (2 articles), brain (2 articles), liver, and kidney (1 article each), and teenagers’ blood and hair (2 articles) (Table 4).

Mercury exposure induces alterations and/or damages to different organs and systems. Most of the observational studies assessed central nervous system injuries (19 articles). Among these, 11 articles investigated the effect of prenatal exposure to methylmercury on neurodevelopment in children (7 h–9 years old) (Table 4).

Morphofunctional changes in neurotransmitters and neuronal cell populations, changes in children's intellectual functions, and possible associations between exposure to mercury and autism spectrum disorders have been observed in the nervous system. Among the selected studies, mercury levels of 0.55 μg/g in maternal hair were associated with impaired neurodevelopment. In addition, Guallar et al. (2002) [38] and Salonen et al. (1995) [39] evaluated the relationship between increased cardiovascular risk and mercury levels after supplementation with docosahexaenoic acid obtained from fish contaminated with mercury (Table 4).

Experimental studies were performed in cell cultures (3 articles) and animal model (5 articles), two in birds, two in fish, and one in rodents. In an animal model, the damage caused by methylmercury (4 articles) and mercury chloride (3 articles) was investigated, with methylmercury administered orally and intraperitoneally, while mercury chloride was diluted in water. Three studies conducted postnatal evaluations, and one did not report the period of exposure, with variations between studies in terms of administered doses, exposure time and mercury concentration (Table 5). Only three animal studies measured mercury concentration. In fish, it was assessed in the gills (1 article), liver (1 article), kidneys (1 article), muscle (2 articles), and brain (2 articles). In birds, the measurements were conducted in blood, feathers, and eggs (1 article).

**Table 5:** Knowledge maps of the experimental study.

Author/years	Model experiments (Animal)	Chemical form of mercury/Exposure pathway	Exposure period	Dose	Time of exposure	Mercury concentration	Evaluated effect
Ganther et al., 1972 [126]	Animal (*Coturnix japonica*)	Methylmercury/Orally	Postnatal (1st–48th day old)	0.83–1.01ppm; 10ppm; 20ppm	48 days	Not reported	Dietary intake of 0.3–0.6 ppm of selenium increased the survival of animals with high concentrations of mercury
Preston et al., 1993 [131]	Cell culture	Mercury chloride	Not reported	0.30 mM	3 days	Not applicable	The cysteine residue 189 is sensitive to mercury ions (Hg^2+^), which can change the osmotic gradient of the cell
Ali et al., 1992 [134]	Animal (Mice/rats) and Cell culture	Methylmercury/Intraperitoneal	Not reported	10–20 μM	Not informed	Not applicable	Increased formation of reactive oxygen species
			Not reported	1 mg/kg (Mice); 5 mg/kg (Rats)	1 day	Not reported	Significant increase in the formation of reactive oxygen species in the cerebellum of mice and rats after 48 h and one week of exposure to methylmercury
Carvalho et al., 2008 [4]	Cell culture	Mercury chloride and methylmercury	7, 15 and 24 h after cell confluence reached ∼70%	10 nM; 25 nM (mercury chloride or methylmercury)	24, 48, and 72 h (Cell growth); 7, 15, and 24 h (TrxR activity)	Not applicable	Mercury chloride has a greater inhibitory effect on the thioredoxin system compared to methylmercury, this effect is rapid and independent of time
Barboza et al., 2018 [150]	Animal (*Dicentrarchus labrax*)	Mercury chloride/Adding mercury to water	Postnatal (juvenile fish)	0.009 mg/L; 0.016 mg/L	96 h	Total mercury (mean) Dose of exposure: 0.009 mg/L - Brain: 0.067 μg/g, muscle: 0.404 μg/g Dose of exposure: 0.016 mg/L - Brain: 0.073 μg/g, muscle: 0.441 μg/g	Inhibition of cerebral acetylcholinesterase, increased lipid peroxidation in brain and muscle, alteration of the activities of energy-related enzymes lactate dehydrogenase and isocitrate dehydrogenase
Evers et al., 2008 [156]	Animal (*Gavia immer*)	Methylmercury/Environmental and feed	Not informed	Not informed	Not informed	Total mercury (mean) Blood: 1.73 μg/g, feather:16.7 μg/g, egg: 1.63 μg/g	Aberrant incubation behavior, lethargy, wing area asymmetry, and reduced reproductive success
Elia et al., 2003 [86]	Animal (*Ictalurus melas*)	Mercury (not specified)/Adding mercury to water	Postnatal (Not reported)	35 μg/L; 70 μg/L; 140 μg/L	10 days	Total mercury (mean) Dose of exposure: 35 μg/L - Gills: 10.73 μg/L, liver: 6.42 μg/L, kidneys: 11.78 μg/L, muscle: 0.74 μg/L Dose of exposure: 70 μg/L -Gills: 10.22 μg/L, liver: 4.82 μg/L, kidneys: 9.82 μg/L, muscle: 0.6 1 μg/L Dose of exposure: 140 μg/L-Gills: 15.28 μg/L, liver: 8.06 μg/L; kidneys: 17.82 μg/L, muscle: 0.80 μg/L)	The lower concentration of mercury reduced the level of glutathione, increased the selenium-dependent glutathione peroxidase, glutathione transferase, glyoxalase I and II, with no significant change in selenium-independent glutathione peroxidase and glutathione reductase

**Fig. 7:**
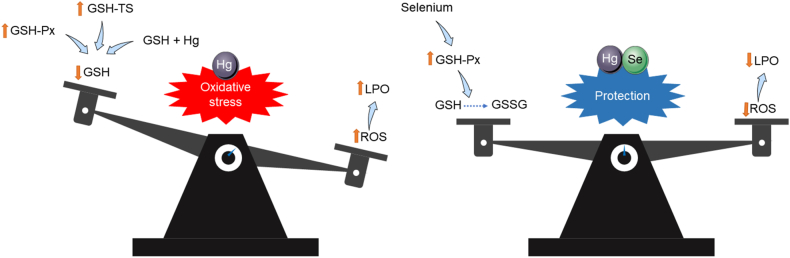
Oxidative damage of mercury and protective effect of selenium. GSH-Px: glutathione peroxidase; GSH-TS: glutathione transferase; GSH: reduced glutathione; GSSG: oxidized glutathione; ROS: reactive oxygen species; LPO: lipid peroxidation.

The effects of mercury described in the studies were increased reactive oxygen species concentrations, a reduction in the level of reduced glutathione (GSH), an increase in selenium-dependent glutathione peroxidase, glutathione transferase, and glyoxalase I and II, an increase in lipid peroxidation, an inhibitory effect on the thioredoxin system (the chloride of mercury has a greater effect than methylmercury), reduced reproductive success, inhibition of brain acetylcholinesterase, and sensitivity of cysteine residue 189 to mercury ions (Hg^2+^). In addition, it demonstrated evidence of the protective effect of selenium (Table 5) (Fig. 7).

## 4 Discussion

This study mapped knowledge from the 100 most-cited articles on the toxic effects of mercury. The studies were published between 1965 and 2021, predominantly as review articles on the mechanisms of mercury-induced damage, such as oxidative stress, and its effects on human and animal health. The selected articles showed that exposure to mercury mainly occurred through feeding. Prenatal exposure to methylmercury was extensively studied, especially its effect on neurodevelopment, and oxidative stress was the most frequently addressed mechanism of damage. The bibliometric analysis herein used allows identifying the trends in a specific study field through the processing, management, and screening of information [40]. It is thus a valuable auxiliary tool for scientists in research planning and for policy-makers. The search was conducted in the WoS-CC database, which indexes high-quality academic journals, allowing the retrieval of a large number of citations from peer-reviewed articles dating back to 1945 [41,42].

This study mapped information from articles highly disseminated by the scientific community and that significantly influenced the knowledge on mercury toxicity. Furthermore, the main laws of bibliometrics, including Bradford's Law, Loka's Law, and Zipf's Law, were applied. The most cited article in this research field was a comprehensive review of the occurrence, exposure, dosage, and health effects of mercury, with insights into other heavy metals such as cadmium, arsenic, and lead, which significantly enriched the understanding of the toxicology of heavy metals [37]. The journal “Environmental Health Perspectives” had the highest number of articles published and cited, suggesting that it is a primary source for research on the topic, according to Bradford's Law [43].

Clarkson TW was the author with the highest number of articles (n = 10) and citations (7116 citations). His study focused on metal toxicology, especially of mercury. He played an important role in the Seychelles Child Development Study, a collaborative project involving the Ministries of Health and Education of Seychelles, University of Rochester, and University of Ulster, that monitored the effects of exposure to mercury in infants and young children, particularly in relation to neurodevelopmental disorders. The articles by Clarkson selected for this study reviewed the toxicity of mercury, including exposure pathways, health effects, and risks [[44], [45], [46], [47]], dose-response relationships [48], and protective mechanisms against mercury toxicity [6]. Clarkson also contributed to a report on methylmercury poisoning in Iraq and its clinical manifestations in fetuses and infants [49], a clinical evaluation study of the effects of this disaster [11], and cohort studies related to prenatal methylmercury exposure and its effects on neurodevelopment [50,51]. Grandjean P (n = 10 articles; 5797 citations) also contributed significantly to mercury toxicology, carrying out research mainly on methylmercury neurotoxicity [16,[52], [53], [54], [55], [56], [57], [58], [59]]. Other authors with relevant contributions include Aschner M, who investigated the uptake of mercury, mainly MeHg, across the blood-brain barrier, its distribution in the brain (neurons and glia), and neurotoxicity mechanisms.

Most corresponding authors of the articles were from the United States (51 authors, 32,673 citations). Authors from North America predominated (58 authors), followed by Europe (26 authors). Although Asia has the highest anthropogenic atmospheric mercury emissions [60], approximately 30% of the mercury present on the ocean surface originates from North America and Europe, with increasing mercury emissions during the 19th and 20th centuries [61].

Mercury is found in various chemical forms with particular toxicokinetic and toxic effects in different tissues and organs [15,18,62,63]. Methylmercury was the most studied chemical form (60% of the observational studies and 57.14% of the experimental studies) and was the second most frequent keyword. Methylmercury is an organic species that is easily absorbed in the intestine [64], and its bioavailability is 3–10 times greater than that of inorganic mercury species [62], it has a strong affinity with thiol (-SH) and selenol (-SeH) groups, and in biological systems most of MeHg+ is linked to low molecular weight compounds (such as cysteine or reduced glutathione) or to –SH or -SeH groups of high molecular weight proteins. Molecular weight [65,66], these characteristics favor absorption and regulate toxicokinetic properties [67], due to these characteristics MeHg^+^ is found in the muscle tissue of fish and rice, being the source of poisoning [67]. Therefore, it poses a greater health risk than other chemical forms and is therefore the most studied chemical form.

In the selected studies, food was the main source of mercury exposure, mainly through the ingestion of fish, seafood, whale meat, and bread with contaminated wheat. The dietary route was predominant, as most studies have evaluated methylmercury, a more stable mercury species with greater bioaccumulation potential than other chemical forms [37,68], mainly in fish and seafood, which are the main sources of human exposure to mercury [69].

The populations studied were from areas with environmental incidents and/or disasters, such as Minamata Bay (Japan), Iraq, the Faroe Islands, and the Republic of Seychelles. Studies with area populations of environmental disasters are relevant because they awaken the need to understand the effect of mercury on human health. The accident in Minamata Bay in the 1950s was the first record of prenatal exposure and alterations in the human brain. Children showed paralysis, intellectual disability, growth, and intelligence changes [8,9,70], emphasizing the effects of prenatal exposure to methylmercury on neurodevelopment. In Iraq in 1970, acute exposure due to the ingestion of bread made with contaminated wheat demonstrated the dose-dependent effects of methylmercury, with doses of 200 mg leading to death [70]. In 1977, elevated levels of mercury were detected in pilot whale meat, which constituted the food base of the population of the Faroe Islands, leading to monitoring and changes in the recommendations for the consumption of this type of meat. In the early 1980s, monitoring of exposure to mercury during pregnancy was initiated in the Republic of Seychelles, a region known for its substantial fish consumption [71].

Although the selected studies addressed events that occurred in the 1950s and the 1970s, concerns about mercury contamination are current and frequent because mercury can travel long distances via air or ocean routes [72]. Artisanal and small-scale mining is the form that most contributes to the levels of mercury in the air; it represents 2/3 of the anthropogenic mercury found in the oceans, 78.5% of all mercury emissions in South America originate in the Amazon [14,15], and this form of contamination is estimated to emit 200 tons of mercury annually [73]. In this context, several riverside populations are vulnerable because their diets are based on fish obtained from the Amazon River and its tributaries contaminated with mercury [12,13] resulting from gold mining. Despite the relevance of this form of contamination in the selected articles, there is a lack of papers specifically addressing the exposure and health risks faced by the Amazonian environment and population, along with strategies for risk mitigation. The prenatal period was the most investigated period of exposure to mercury (approximately 75% of the observational studies), and it is vulnerable to the effects of toxins or other agents [51]. The placental barrier is ineffective in protecting the fetus from mercury [51], especially from organometallic complexes formed by the binding of MeHg + to compounds containing thiol (-SH) and selenol (-SeH) groups, but specifically L-Cys, which forms the L-Cys-S-MeHg complex that mimics l-methionine and crosses the placental barrier and blood-brain barrier through L-type large neutral amino acid transporters (LATs) [65,66] mainly organic mercury and alters fetal development [15]. Mercury vapor also crosses the placenta, but the passage of inorganic mercury tends to be more limited than that of organic mercury [34,74]. Owing to its toxicokinetic characteristics, methylmercury primarily targets the central nervous system [75].

Most of the articles involving humans quantified mercury in more than one matrix. In studies that evaluated prenatal exposure, mercury levels were quantified in maternal milk (5% of the articles), the umbilical cord (blood, serum and tissue; 35%, 5% and 10% respectively), maternal and/or infantile hair (60 % of the articles) and blood (20% of the articles), brain (10% of the articles), liver (5% of the article), and kidney (5% of the article) of children, and postnatal exposure to mercury levels were measured in toenails (5% of the articles), hair (5% of the articles), and urine (5% of the articles) (Table 4). The choice of adequate fluid or tissue is important for assessing the scenarios of exposure to mercury [75]. Blood is commonly used for analyzing recent exposure to organic mercury, urine for inorganic mercury [75], and hair for long-term exposure of organic mercury [75,76]. Toenails grow slowly (12–18 months) and may be a stable specimen after long-term exposure to toxic metals, including methylmercury [[77], [78], [79]]. This matrix may reflect exposure in the previous 3–5 [80] or 6–9 months [81] and is easier to collect, transport, and store than other matrices [79]. Nails can be an alternative for isolated populations and for men with short hair [82]. However, there is no standardized method for the collection and analysis of samples [83]. Zhang et al. (2022) [82] suggested that fingernails are less sensitive to methylmercury, inorganic mercury, and total mercury, whose concentration and excretion values are lower than those of hair by 2–4 times and 70–226 times, respectively. Other studies have showed that total mercury levels in toenails correlate with brain and blood methylmercury levels [84], and nail mercury levels correlate with hair levels with a slope of 2.79 for hair vs. toenail levels [85].

There is a correlation in the ratio of 250:1 in the concentration of mercury in capillary and whole blood, with maternal hair proving to be a reliable biomarker in prenatal exposure, as it correlates with mercury levels in fetal blood [75]. This proportion is used by the World Health Organization (WHO) to convert capillary concentration (μg/kg) into blood concentration (μg/L) [75,76], but this may vary according to the population and have dietary influence [76].Umbilical cord blood can be used to safely and accurately infer mercury levels in newborns after exposure [57]. The levels of this metal in the kidneys have been measured because of its accumulation and consequent damage [86,87] [86](86)(86). The liver is another organ of great interest for being rich in compounds containing thiol groups (glutathione and thioredoxin) that play an important role in the homeostasis of the redox system and bind to thiols, causing the loss and/or reduction of antioxidant function; this may trigger an imbalance in the redox system and result in hepatic and systemic oxidative damage [88].

Most prenatal exposure studies have consistently indicated neurobehavioral impairments in children related to motor and cognitive functions, such as memory, attention, language, and deficiencies in visuospatial perception. However, notable inconsistency and variation arise when considering the exposure levels required to trigger such neurodevelopmental effects. Several studies have highlighted this disparity, presenting mercury values in different biological compartments that vary considerably. For example, studies reporting changes in memory have identified maternal hair mercury values of 0.55 μg/g [89], 4.27 μg/g [16], and 12.50 μg/g [16,54]. Mercury values in children's hair were recorded at 2.99 μg/g [54], while in umbilical cords, these values varied between 22.90 μg/L [54] and 59.00 μg/L [54]. Similar variability is observed in studies related to changes in attention, with mercury values in maternal hair recorded at 4.27 μg/g [16] and 4.21 μg/g [57]. For children's hair, the values were 2.99 μg/g [16] and 4.21 μg/g [57], while in adolescents, both in hair and blood, values were observed of 0.96 μg/g and 4.08 μg/L, respectively [57]. This substantial variation, as evidenced by the studies cited, highlights the complexity of understanding the impacts of prenatal mercury exposure on neurological development. These discrepancies can be attributed to differences in the populations studied, analytical methodologies, and environmental factors [76].

Organic mercury easily binds to sulfhydryl groups, facilitating its passage through the blood-brain barrier, leading to accumulation in the central nervous system [90] in the development and/or maturation phase, causing enzymatic and metabolic disturbances [91] and consequent brain development impairment [90] through disruption in plastic processes such as cell migration, differentiation, synaptogenesis, myelination, and apoptosis [92]. Changes during this period lead to irreversible compromise of the developing brain, with lasting effects on learning and memory [92].

**Fig. 8:**
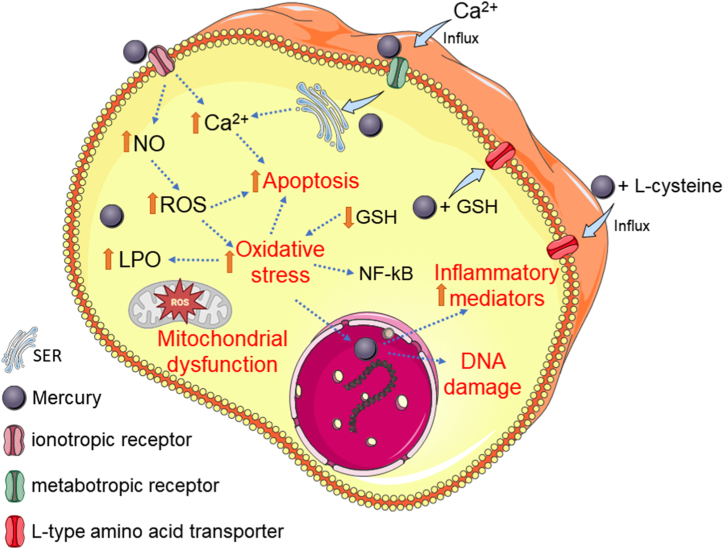
Mechanism of the mercury-induced cell damage. SER: sarcoplasmic endoplasmic reticulum; ROS: reactive oxygen species; LPO: lipid peroxidation; NO: nitric oxide; GSH: reduced glutathione; NF-kB: factor nuclear kappa B; Ca^+2^: calcium ions.

The damage mechanisms induced by exposure to mercury are multifactorial, and oxidative and inflammatory changes were most frequently reported by the selected studies, with “oxidative stress” being the third most frequent keyword. These oxidative alterations occur because of an imbalance in the redox system, which can occur due to the depletion of antioxidants and/or an increase in the formation of free radicals [93]. The depletion of antioxidants occurs mainly because of the influx of GSH linked to mercury and/or glutathione oxidation in the repair of biomolecules oxidized by reactive species, in addition to interference with the activity of several proteins and/or enzymes in antioxidant defense, including thioredoxin, glutaredoxin, glutathione peroxidase, glutathione reductase, thioredoxin reductase, and superoxide dismutase [67,70,93,94] (Fig. 8).

An increase in reactive species occurs by activating the nitric oxide (NO) synthesis pathway, leading to an increase in reactive oxygen and nitrogen species [95]. These mechanisms together provide an imbalance in the redox system and consequent oxidative stress. This situation can induce DNA damage, increased expression of proteins that induce the apoptotic process, and inflammatory factors such as NF-kB [96] (Fig. 8).

In addition, mercury increases cytoplasmic calcium ion levels by increasing extracellular calcium influx and releasing calcium from the sarcoplasmic endoplasmic reticulum, which is mediated by signaling pathways triggered by the activation of metabotropic receptors [97]. This increase in cytoplasmic calcium ion levels can increase the expression of proteins that induce apoptosis and/or mitochondrial dysfunction [98]. In addition, it interferes with the synthesis, frelease, and function of the neurotransmitters GABA, glutamate, serotonin, dopamine, and acetylcholine [70,99] (Fig. 8).

Recent studies have also described damage to the reproductive system, changes in fertility and during pregnancy [100,101], ophthalmological damage, such as visual color changes sensorimotor polyneuropathy and mononeuropathy multiplex [102], hepatotoxicity [88,103], association with increased risk of urinary tract cancer [104], damage to the DNA and proteomic profile of salivary glands [20], metabolic and oxidative alterations in salivary gland cells [105], changes in salivary parameters [106], and damage to alveolar bone tissue [25]. Recent studies have focused on the neuroprotective effect of apigenin, a flavonoid with antioxidant and anti-inflammatory properties [107], the antioxidant action of unsaturated fatty acids from *Pteleopsis suberosa* stem bark extract on mercury-induced testicular dysfunction [108] and the neuroprotective action of açaí (*Euterpe oleracea, Mart*.) [109].

Mapping the knowledge on the effects of exposure to mercury is crucial to public health as it allows the identification of “hot topics,” dynamic trends, current advancements, and the unexplored scientific domains within this subject matter. Bibliometric analysis is limited by its lack of ability to detect recently published articles with a low number of citations and by the inflation of number of citations resulting from self-citation. However, it is important for understanding the landscape of scientific mercury production. The results of the present study can thus serve as a basis for new clinical, epidemiological, and experimental studies aimed at enhancing the understanding of the mechanisms of damage in underexplored structures/organs and tissues, as well as the mechanisms of protection against this damage.

Studies in humans primarily addressed the effects of acute exposure to methylmercury resulting from environmental disasters, did not address the difference between sexes, and most studies focused on changes in the central nervous and cardiovascular systems. Studies in animal models have evaluated the protective effects of selenium. These results demonstrate important gaps in knowledge that still need to be better understood, such as changes in the stomatognathic system and blood cells, as well absence of less invasive techniques such as saliva and lack of correlation between mercury levels and scale of diagnosed damage. Additionally, it was shown that studies investigating longitudinal biomonitoring, especially of populations subjected to low doses for a prolonged period are necessary, in addition to investigating pharmacological and non-pharmacological alternatives to mitigate and/or avoid damage induced by mercury, among the non-pharmacological alternatives the use of natural products as modulators of the redox system to protect against oxidative stress triggered by mercury.

## 5 Conclusion

This study identified and analyzed the 100 most-cited articles on biological impairments caused by mercury in animal experimentation and/or diagnosis in humans. These studies mostly evaluated prenatal and postnatal exposure to different doses of mercury that affected neurodevelopment and neurobehavior in children, in addition to endocrine and cardiovascular changes. Oxidative stress was the most discussed damage mechanism, and some articles addressed the protection against this damage.

Furthermore, it demonstrated the need for investigations to better understand gaps in knowledge, such as in pharmacokinetic aspects and the correlation of mercury levels with damage, as well as the use of less invasive alternative techniques such as the evaluation of saliva to measure mercury and its impacts. Understanding the mechanisms of damage and de exposure threshold can serve as a foundation for the guidelines and policies of governmental and non-governmental organizations.

## Funding

P.F.S.M. and L.O.B. received a scholarship from Fundação Amazônia de Amparo a Estudos e Pesquisas (10.13039/501100005288FAPESPA); V.S.C received a scholarship from Coordenação de Aperfeiçoamento de Pessoal de Nível Superior (10.13039/501100002322CAPES – Finance Code 001). R.R.L is a researcher from Conselho Nacional de Desenvolvimento Científico e Tecnológico (10.13039/501100003593CNPq) and received a grant under number 312275/2021-8 and 408329/2022-0. M.A. was supported in part by a grant from the 10.13039/100000066National Institute of Environmental Health Sciences (NIEHS) under the number R01ES07331. The APC was funded by Pró-Reitoria de Pesquisa e Pós-graduação from Federal University of Pará (PROPESP)

## Data availability

All data are reported in the manuscript and in references therein.

## CRediT authorship contribution statement

**Daiane Claydes Baia-da-Silva:** Writing – original draft, Software, Methodology, Investigation, Formal analysis, Conceptualization. **Paulo Fernando Santos Mendes:** Software, Methodology, Investigation. **Diane Cleydes Baia da Silva:** Software, Methodology, Investigation. **Victória Santos Chemelo:** Visualization, Validation, Formal analysis. **Leonardo Oliveira Bittencourt:** Writing – review & editing, Visualization, Validation, Formal analysis. **Pedro Magalhães Padilha:** Writing – review & editing, Visualization, Validation, Formal analysis. **Reinaldo Barreto Oriá:** Writing – review & editing, Visualization, Validation, Formal analysis. **Michael Aschner:** Writing – review & editing, Visualization, Validation, Formal analysis. **Rafael Rodrigues Lima:** Writing – review & editing, Visualization, Validation, Supervision, Software, Resources, Project administration, Methodology, Investigation, Funding acquisition, Formal analysis, Data curation, Conceptualization.

## Declaration of competing interest

The authors declare that they have no known competing financial interests or personal relationships that could have appeared to influence the work reported in this paper.
